# Elevated APOBEC mutational signatures implicate chronic injury in etiology of an aggressive head-and-neck squamous cell carcinoma: a case report

**DOI:** 10.1186/s13256-021-02685-w

**Published:** 2021-04-30

**Authors:** Jena Patel, Nicoline Y. den Breems, Madalina Tuluc, Jennifer Johnson, Joseph M Curry, Andrew P. South, Raymond J. Cho

**Affiliations:** 1 Department of Otolaryngology–Head and Neck Surgery, Thomas Jefferson University, 925 Chestnut St, Philadelphia, PA 19801 USA; 2 Centre for Advanced Computational Solutions (C-fACS), Faculty of Agriculture and Life Sciences, Lincoln University, Lincoln, 7647 Canterbury New Zealand; 3 Department of Pathology, Anatomy and Cell Biology, Thomas Jefferson University, Philadelphia, PA 19107 USA; 4 Department of Oncology, Thomas Jefferson University, Philadelphia, PA 19107 USA; 5 Department of Dermatology and Cutaneous Biology, Thomas Jefferson University, Philadelphia, PA 19107 USA; 6 Department of Dermatology, University of California, San Francisco, CA 94115 USA

**Keywords:** APOBEC, Squamous cell carcinoma, RDEB, Epithelial injury

## Abstract

**Background:**

Aggressive squamous cell carcinomas (SCCs) present frequently in the context of chronic skin injury occurring in patients with the congenital blistering disease recessive dystrophic epidermolysis bullosa. Recently, these cancers were shown to harbor mutation signatures associated with endogenous deaminases of the active polynucleotide cytosine deaminase family, collectively termed APOBEC, and clock-like COSMIC [Catalogue of Somatic Mutations in Cancer] signatures, which are associated with normal aging and might result from cumulative DNA replication errors. We present a case of a nasal septal SCC arising in the context of recurrent injury, but also modest past tobacco use. Our genetic analysis of this tumor reveals unusually high APOBEC and clock-like but low tobacco-related COSMIC signatures, suggesting that chronic injury may have played a primary role in somatic mutation. This case report demonstrates how signature-based analyses may implicate key roles for certain mutagenic forces in individual malignancies such as head-and-neck SCC, with multiple etiological origins.

**Case presentation:**

We report the case of a 43-year-old male former smoker who presented with congestion and swelling following a traumatic nasal fracture. During surgery, the mucosa surrounding the right nasal valve appeared abnormal, and biopsies revealed invasive keratinizing SCC. Frozen section biopsies revealed multiple areas to be positive for SCC. Gene sequencing showed loss of *PTEN* (exons 2–8), *CDKN2A/B* and *TP53* (exons 8–9), *MYC* amplification, and BLM S338*. Exome sequencing data also revealed that 36% of mutations matched an APOBEC mutational signature (COSMIC signatures 2 and 13) and 53% of mutations matched the clock-like mutation signature (COSMIC signature 5). These proportions place this tumor in the 90th percentile bearing each signature, independently, in a reference data set combining cutaneous and The Cancer Genome Atlas (TCGA) head and neck SCC data. In contrast, few mutations harbored a tobacco-related COSMIC signature 4, representing about the 10th percentile in our reference SCC data set. The patient was treated with partial rhinectomy with local flap reconstruction, bilateral neck dissection, and adjuvant radiation therapy; the patient remains disease-free to date.

**Conclusion:**

Based on comparative mutational signature analysis, we propose that the history of tobacco use and traumatic injury may have collaborated in activating APOBEC enzymes and the clock-like mutational process, ultimately leading to cancer formation. Clinical awareness of the relationship between epithelial injury and tumorigenesis should enhance earlier detection of this particularly aggressive type of cancer.

## Background

Cutaneous squamous cell carcinomas (SCCs) sometimes arise with chronic epithelial injury, such as in patients with recessive dystrophic epidermolysis bullosa (RDEB). These skin cancers behave more aggressively than ultraviolet light (UV)-derived skin cancers [1]. Recently, cutaneous SCCs from chronically injured epithelium of RDEB patients were shown to harbor somatic mutation patterns associated with enhanced activity of the apolipoprotein B mRNA-editing enzyme catalytic polypeptide–like (APOBEC) cytosine deaminases (determined by their DNA context rather than the genes in which they arise), also known in aggregate as “mutation signatures” [1, 2]. These findings suggest that chronically injured cells undergo a predictable form of mutagenesis, and that epithelial cancers bearing this signature deserve close monitoring. We genetically characterized a metastatic head-and-neck SCC of the nasal septum with clinical symptoms of long-term injury. Exome sequencing revealed a sharply enhanced APOBEC mutation signature, supporting a model in which these mechanisms cause aggressive skin cancers originating in chronic wounds. To our knowledge, this is the first case report linking specific mutational signatures with head-and-neck SCC arising at a site of chronic injury. Such cancers should be monitored more closely, and potentially clinically evaluated for early dissemination.

## Case presentation

A 43-year-old Caucasian man presented with a traumatic nasal fracture. He noted difficulty breathing and soreness at the right side of the nose, and a 5-year history of mild tenderness at the same site. On physical exam, his nose was deviated to the right, and nasal endoscopy revealed normal-appearing mucosa with some anterior crusting. The patient had a 7.5-pack-year smoking history, with cessation 13 years earlier. The patient did not have any significant medical history otherwise and did not have a family history of cancer.

**Fig. 1:**
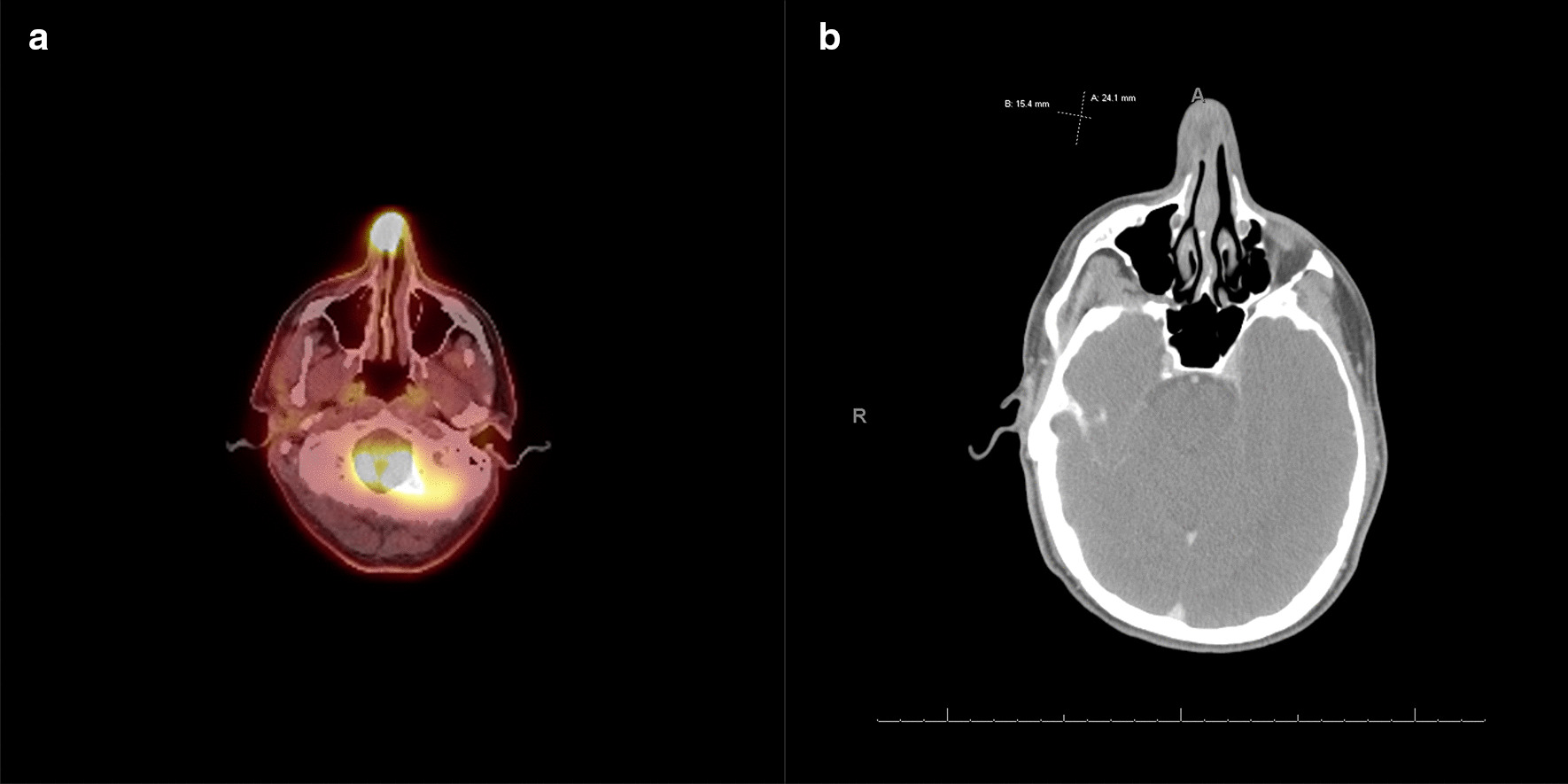
Patient imaging findings including head and neck **a** positron emission tomography–computed tomography (PET-CT) and **b** CT with contrast showing 15.4 mm × 24.1 mm right nasal mass and local disease only

During repair of the fracture, the right external nasal valve mucosa appeared ulcerated. Biopsy revealed invasive keratinizing SCC with perineural involvement. Subsequent computed tomography (CT) and positron emission tomography (PET)/CT imaging showed local disease only (Fig. 1). The patient underwent partial rhinectomy with a paramedian forehead flap reconstruction; negative histopathologic margins were established. The lesion spanned 2.4 cm and involved the right lateral nasal wall with extension to the septum and invasion through the cartilage into the subcutaneous tissues of the right ala. On 2-month follow-up, the patient reported a new, palpable submandibular lymph node on the right side. Fine needle aspiration showed regionally metastatic disease. Bilateral neck dissection revealed disease in one of 56 excised lymph nodes at level 1B; the lymph node was 3 cm in size. After surgery, the patient had a final pathologic stage of T2N1M0. He underwent adjuvant radiation therapy using volumetric-modulated arc therapy (VMAT) to the nasal cavity and bilateral neck; he received a total of 60 Gy in 30 fractions. The patient tolerated therapy well, with no interruptions or major toxicity. The 5-year overall survival for early-stage T1–T2 disease was estimated at 58–85%; however, due to limited comparison data it was difficult to determine the patient’s exact prognosis. To date, the patient has no evidence of disease recurrence.

**Fig. 2:**
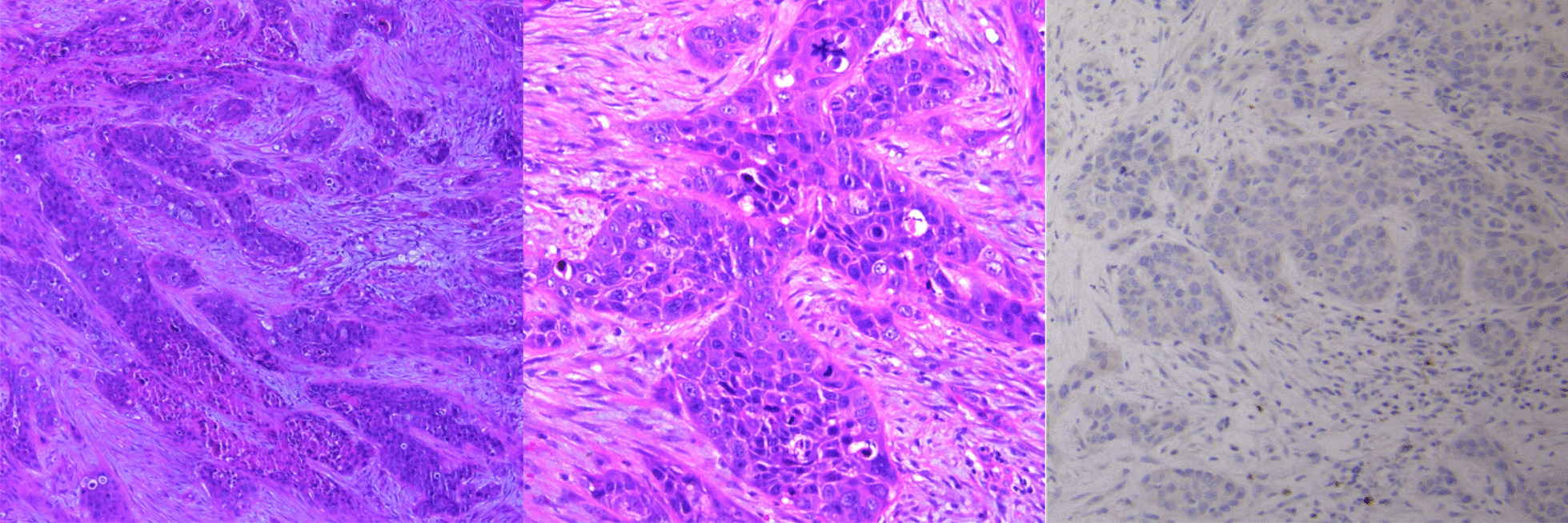
Invasive, p16-negative squamous cell cancer (SCC) of the nasal septum arising in the context of chronic injury. **a** Hematoxylin and eosin staining of SCC (×4 magnification), **b** ×10 magnification, **c** p16 staining demonstrating loss of p16 in tumor (black arrow), with preservation of signal in stroma (red arrow)

**Fig. 3:**
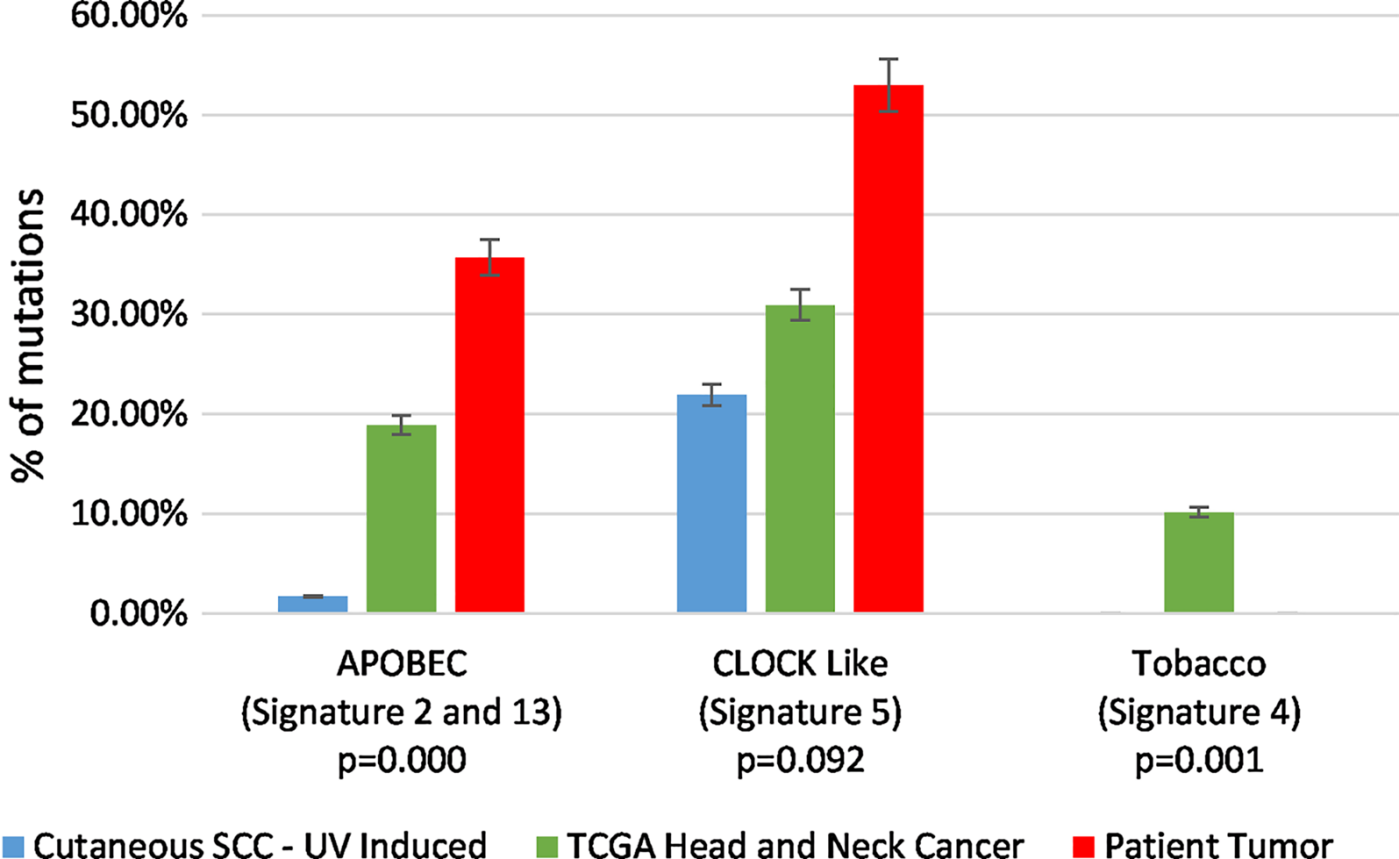
Apolipoprotein B mRNA-editing enzyme catalytic polypeptide–like (APOBEC) mutation signatures are sharply enhanced in a squamous cell carcinoma (SCC) arising in a clinical context of nasal septal injury. Column graph shows percentage of mutations matching each of three stereotyped patterns (“signatures”) [3, 4] of nucleotide mutation and context. Catalogue of Somatic Mutations in Cancer (COSMIC) signatures 2 and 13 (36%) are associated with APOBEC mutagenesis and are detected on average at levels <5% in spontaneous, ultraviolet (UV)-derived cutaneous SCCs. COSMIC signature 4 is associated with UV light exposure and mutagenesis. COSMIC signature 5 represents a less specific mutagenesis pattern found in many cancer types (clock-like) and associated with normal aging, as well as chronic tissue injury [3]. *p *values show results of a one-way analysis of variance assessing whether the prevalence of a mutation signature is significantly different in the patient’s tumor (red) compared to a cohort of 38 UV-induced SCCs (blue) and 279 The Cancer Genome Atlas (TCGA) head-and-neck SCCs (green). This analysis demonstrated a significant overrepresentation of the APOBEC signature (*p* = 0.000) and underrepresentation of the tobacco signature (*p* = 0.001) in our patient’s tumor, compared to the reference data set. Mutational signature assignation from exome sequencing data was performed using the deconstructSigs method [5]

Immunohistochemistry of the tumor was positive for cytokeratin and negative for p16 (Fig. 2). Human papilloma virus type 16 (HPV16) and HPV18 were negative. FoundationOne^®^ analysis showed loss of *PTEN* (exons 2–8), *CDKN2A*/*B* and *TP53* (exons 8–9), *MYC* amplification, and a BLM S338* truncating mutation. Exome sequencing on the Illumina HiSEQ platform with >110× nucleotide coverage [1] revealed 435 somatic single-nucleotide variants. Thirty-six percent of mutations matched an apolipoprotein B mRNA-editing enzyme catalytic polypeptide–like (APOBEC) mutational signature (2 and 13), characterized by the presence of C>T substitutions (signature 2) and C>G substitutions (signature 13) (Fig. 3). One-way analysis of variance (ANOVA) showed a significant overrepresentation of the APOBEC signature (*p* = 0.000) and underrepresentation of the tobacco-related signature (*p* = 0.001) in the nasal septal squamous cell carcinoma (SCC), compared to the reference SCC data set. There was no ultraviolet (UV)-associated COSMIC signature 7 (associated with large numbers of CC>TT dinucleotide mutations) or tobacco-associated Catalogue of Somatic Mutations in Cancer (COSMIC) signature 4 (characterized by CC>AA dinucleotide substitutions); however, there was a substantial COSMIC signature 5 (characterized by transcriptional strand bias for C>T substitutions at ApTpN context) contribution (53%).

**Fig. 4:**
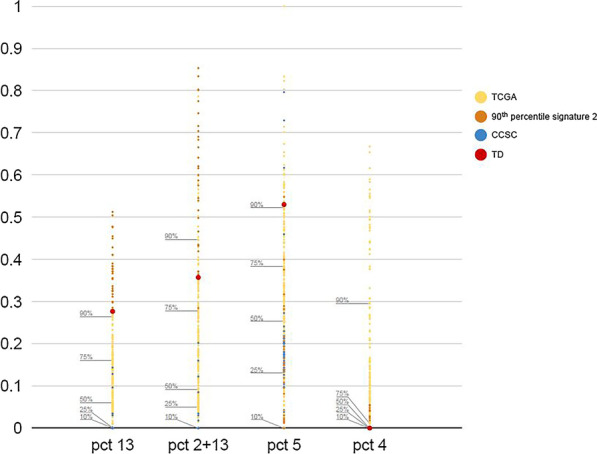
Unusually elevated proportions of apolipoprotein B mRNA-editing enzyme catalytic polypeptide–like (APOBEC) and clock-like mutagenesis in a nasal septal squamous cell carcinoma (SCC). The *y*-axis represents the percentage of mutations in a given cancer attributable to COSMIC signature 2 (APOBEC), signatures 2 and 13 (APOBEC subset), signature 5 (clock-like), and signature 4 (tobacco), which are shown on the *x*-axis. The dots represent 279 The Cancer Genome Atlas (TCGA) head-and-neck SCCs (yellow, except for 27 tumors in the 90th percentile for signature 2 proportion, represented in brown), 38 cutaneous SCCs (blue), and our nasal septal SCC (red). Our patient’s nasal septal SCC is in the 90th percentile for both COSMIC signatures 2 and 5 independently, and in the 80th percentile for signatures 2 and 13. The SCCs in the 90th percentile for signature 2 (brown, first column) show good concordance with elevated COSMIC signature 2 and 13 proportions (overall APOBEC, second column), but not with COSMIC signature 5 (clock-like , third column). Therefore, the fact that our tumor scores highly in both APOBEC and clock-like signatures is unusual, and more closely resembles the tissue damage SCCs reported in recessive dystrophic epidermolysis bullosa [1].

Notably, these data place mutations from our patient’s tumor matching an APOBEC mutational signature 2 and those matching a clock-like mutation COSMIC signature 5 each in the 90th percentile of a reference data set consisting of The Cancer Genome Atlas (TCGA) head-and-neck SCCs and a separate data set of cutaneous SCCs (Fig. 4). The top 90th percentile of *APOBEC* signature 2-containing TCGA head-and-neck SCCs (shown in brown) are highly represented in the 90th percentile of APOBEC COSMIC signature 13-containing tumors, confirming their expected concordance (Fig. 3). In contrast, few of the reference APOBEC COSMIC signature 2-elevated group appear in the top 90th percentile of clock-like COSMIC signature 5 mutations, as do our patient’s tumor and like those injury-derived SCCs recently reported to arise in an RDEB background [1]. Notably, the patient’s tumor also did not harbor any tobacco-related COSMIC signature 4 mutations, placing it in about the 10th percentile in the reference data set.

## Discussion

SCC can arise from chronic injury, initiated by processes ranging from bullous disease to burn injury, a process recently linked to the APOBEC nucleic acid editing system. Physiologically, APOBEC enzymes possess cytosine deaminase activity for RNA and single-stranded DNA (ssDNA), facilitating antibody diversification and lipid metabolism, and protecting the host from viral infection [2]. However, whole genome sequencing has demonstrated that these deaminases can be a major source of clustered nonrandom genomic mutations in multiple types of cancers including prostate and colorectal cancer [1, 2, 6].

Our patient’s tumor harbored approximately seven mutations per megabase (mB), similar to RDEB cSCCs and below the >100 mutations/mB observed in UV-driven cSCCs. Relative to most head-and-neck SCC and cutaneous SCC, this cancer harbors an elevated percentage of APOBEC COSMIC signatures 2 and 13 and clock-like COSMIC signature 5 mutations. Although the patient was a former smoker, there were no COSMIC signature 4 mutations, suggesting that this mechanism was not as active in this tumor. Therefore, we speculate that tumors like our patient’s, which harbor larger proportions of APOBEC and clock-like mutations relative to tobacco-associated mutations, arise from a source of chronic injury or inflammation other than tobacco. The patient’s extended clinical history of soreness at this site suggests a possible source of injury preceding tumor development. This hypothetical link between relative mutation signatures and causative etiology is testable and deserves further evaluation.

While the relative mutation signature interpretations we present here are largely qualitative, in Fig. 3, we develop and present a percentile-based comparative analysis in which our patient’s tumor is assessed in the context of a large reference data set of signatures from similar cancers. This analysis illustrates the rarity of a head-and-neck tumor harboring both elevated APOBEC and clock-like mutations, as seen in the patient profiled here, and lays groundwork for future comparative quantitative analysis of mutation signatures in individual cancers. Future development of APOBEC-inhibitors could offer options where chronic injury is clinically apparent but difficult to treat [7].

A limitation of this case report and our method assessing mutation signatures in individual patients is that we cannot determine whether the initial septal injury was due to trauma or an endogenous inflammatory process; it is also possible that tobacco exposure contributed to re-injury at this site. However, awareness of this relationship between epithelial injury and a specific form of mutagenesis should alter clinical management of SCCs.

## Conclusion

An overrepresented APOBEC mutational signature may predict aggressive cutaneous SCCs, possibly because of parallel, injury-mediated disruption of the microenvironment, warranting thorough surgical extirpation and long-term monitoring. Development of APOBEC inhibitors could serve as a potential therapy in cases involving chronic injury.

## Data Availability

The data sets used and/or analyzed during the current study are available from the corresponding author on reasonable request.

## Abbreviations

SCC: Squamous cell carcinoma
RDEB: Recessive dystrophic epidermolysis bullosa
APOBEC: Apolipoprotein B mRNA-editing enzyme catalytic polypeptide–like
